# Comparison between a Direct-Flow SPR Immunosensor for Ampicillin and a Competitive Conventional Amperometric Device: Analytical Features and Possible Applications to Real Samples

**DOI:** 10.3390/s17040819

**Published:** 2017-04-10

**Authors:** Mauro Tomassetti, Giovanni Merola, Elisabetta Martini, Luigi Campanella, Gabriella Sanzò, Gabriele Favero, Franco Mazzei

**Affiliations:** 1 Department of Chemistry, “Sapienza” University of Rome, P.le A. Moro, 5, 00185 Rome, Italy; gancetto2@hotmail.it (G.M.); elisabettamartini@libero.it (E.M.); luigi.campanella@uniroma1.it (L.C.); 2 Department of Chemistry and Pharmacy Technology, “Sapienza” University of Rome, P.le A. Moro, 5, 00185 Rome, Italy; gabriella.sanzo@uniroma1.it (G.S.); gabriele.favero@uniroma1.it (G.F.); franco.mazzei@uniroma1.it (F.M.)

**Keywords:** ampicillin, flow surface plasmon resonance (SPR) immunosensor, competitive amperometric immunosensor, analytical comparison

## Abstract

In this research, we developed a direct-flow surface plasmon resonance (SPR) immunosensor for ampicillin to perform direct, simple, and fast measurements of this important antibiotic. In order to better evaluate the performance, it was compared with a conventional amperometric immunosensor, working with a competitive format with the aim of finding out experimental real advantages and disadvantages of two respective methods. Results showed that certain analytical features of the new SPR immunodevice, such as the lower limit of detection (LOD) value and the width of the linear range, are poorer than those of a conventional amperometric immunosensor, which adversely affects the application to samples such as natural waters. On the other hand, the SPR immunosensor was more selective to ampicillin, and measurements were more easily and quickly attained compared to those performed with the conventional competitive immunosensor.

## 1. Introduction

Ampicillin is a broad-spectrum, semi-synthetic, beta-lactam penicillin antibiotic with bactericidal activity, used to treat bacterial infections caused by susceptible, usually Gram-positive, organisms. Ampicillin has in vitro activity against Gram-positive and Gram-negative aerobic and anaerobic bacteria. The bactericidal activity of ampicillin results from inhibiting cell wall synthesis and is mediated through ampicillin’s binding to penicillin binding proteins (PBPs). In fact, ampicillin binds to and inactivates penicillin-binding proteins located on the inner membrane of the bacterial cell wall. Inactivation of PBPs interferes with the cross-linkage of peptidoglycan chains necessary for bacterial cell wall strength and rigidity.

This interrupts bacterial cell wall synthesis [1], resulting in a weakening of the bacterial cell wall and causes cell lysis. Ampicillin is stable against hydrolysis by a variety of beta-lactamases including penicillinases, cephalosporinases, and extended spectrum beta-lactamases, and thus can be used in a wide range of Gram-positive and Gram-negative infections [1]. Of course, due to the great importance of antibiotics in the clinical and pharmaceutical fields, several strategies, such as chromatography [2,3,4,5,6], mass spectrometry [7,8,9,10], and microbial screening [11,12], as well as methods based on sensors, biosensors and immunosensors [13,14,15,16,17,18,19,20], have been developed for the analytical determination of ampicillin and other antibiotics. Our research group has devoted several efforts in developing several kind of sensors for different type of antibiotics: for instance, we developed different sensors for β-lactam antibiotic detection, initially ion selective electrodes (ISEs) [21] and most recently amperometric immunosensors [22,23]; these amperometric immunosensors have proven to be very efficient in terms of analytical characteristics (very low LOD, wide linear range, good repeatability, and accuracy) but measurements, of competitive formats, require up to an hour, so analysis time is often too long for the requirements of modern analytical chemistry. Therefore, researchers have been attempting to develop methods for direct instead of competitive measurement to save time. Most of such methods have attempted to achieve this by using more modern transducers, generally of the SPR (surface plasmon resonance) type. This approach generally provides good results when the target molecule has a high molecular weight (proteins and so on). However, when the molecule to be determined has a low molecular weight (as is the case for many drugs), the analytical efficiency of an SPR transducer is significantly reduced. For instance, some researchers in our laboratories have recently attempted to develop an SPR immunosensor [24] to ampicillin based on the sandwich method, but achieved only partial success. Therefore, to better investigate these results, in the present research, we compared a simpler immunosensor for the direct determination of ampicillin using the classical-flow SPR technique and compared its analytical features with a conventional competitive immunosensor device. The two methods were used to analyze ampicillin in different real samples: bovine milk, river water, and spring surface water samples. Ampicillin was chosen for its wide use in the medical and veterinary fields, as it is often found in food samples of biological and environmental interest.

## 2. Materials and Methods

### 2.1. Reagents and Samples

Anti-ampicillin, monoclonal antibody was provided by Acris (Acris Antibodies GmbH, Herford, Germany), while magnesium chloride, potassium chloride, and dibasic and monobasic anhydrous potassium phosphate RPE (Reagents for European Pharmacopoeia) were supplied by Carlo Erba Reagents (Carlo Erba, Milan, Italy). Ny^+^ Immobilion Affinity membrane (porosity 0.65 μm) was provided by Millipore (Millipore Corporation, Billerica, MA, USA). Biotin Tag^TM^ Micro Biotinylation Kit, composed of biotinylation Reagent (BAC-SulfoNHS, namely biotinamido hexanoic acid 3-sulfo-n-hydroxysuccinide ester), 5 M sodium chloride solution, micro-spin column (2 mL) (in practice, a small empty cylindrical vessel prepackaged with Sephadex G-50), 0.1 M sodium phosphate buffer pH 7.2, 0.01 M phosphate buffer saline (PBS) pH 7.4 (reconstituted with 1 L of deionized water to give 0.01 M phosphate buffer, 0.138 M NaCl, 2.7 mM KCl, pH 7.4), and ExtrAvidin^®^ peroxidase (containing 0.2 mL of ExtrAvidin Peroxidase conjugate at 2.0 mg·mL^−1^, with 0.01% thimerosal), Fosfomycin, 6-amino-penicillanic acid, dialysis membrane (art. D-9777), albumin from bovine serum (BSA), TRIS (hydroxymethyl-aminomethane), and TWEEN^®^ 20 were all provided by Sigma (Sigma Aldrich, Milan, Italy). Dicloxacillin, cefotaxime, cefalexin, and potassium penicillin G were provided by IBI (I.B.I. Sud Spa, Milan, Italy). Sodium ampicillin was provided by Farmitalia (Farmitalia, Carlo Erba S.p.A., Milan, Italy). Piperacillin was provided by Lederle (Cynamid Italia, Catania, Italy). Neomycin and bacitracin were produced by Boehringer Ingelheim It Spa (Milan, Italy). Ampicillin was produced by Fluka Analyticals (St. Louis, MO, USA); fosfomycin was produced by Crinos S.P.A. (Villa Guardia, Como, Italy). Sodium dihydrogen phosphate NaH_2_PO_4_ (≥99.0%), sodium dibasic phosphate Na_2_HPO_4_ (≥99.0%), EtOH (96%), 11-mercaptoundecanoic acid (MUA) (95%), 1-ethyl-3-(3-dimethyl-aminopropyl) carbodiimide (EDC), N-hydroxysuccinimide (NHS) (98%), glycine, ethanolamine (≥99%), ceftriaxone, and erythromycin were supplied by Sigma-Aldrich (St. Louis, MO, USA). The SPR plates, each of which was composed of a layer of Au with a thickness of 50 nm on a glassy support, were supplied by XanTec bioanalytics GmbH (Dusseldorf, Germany). The oil with a refractive index of 1.6100 ± 0.0002 was supplied by Cargille Laboratories (Cedar Grove, NJ, USA).

Real analyzed samples were as follows: cow milk, derived from a farm in Central Italy (Lazio); water samples from Sacco River (Lazio); and spring surface water near Rome. In the case of the amperometric method, 0.5 mL of each sample were added to 5 mL of a buffer, and this solution was then subjected as is for analysis. In the case of the SPR method, only a simple dilution with the buffer was necessary; in particular, 0.5 mL of each sample were added to 0.5 mL of the buffer and analyzed.

### 2.2. Flow SPR or Amperometric Apparatus

SPR flow measurements were performed by using a BioSuplar 400T apparatus (Analytical μ-Systems-Department of Mivitec GmbH, Sinzing, Germany), with a laser diode with low power (630–670 nm) as a light source. This instrument allows for the quantitative analysis of molecules on the basis of the mass of the antibody complex on the plate, which produces a variation successively processed as a function of time in the form of a sensorgram. For ampicillin analysis, using the conventional immunosensor, a 551 VA-Detector Amel potentiostat was used, connected to a 4006a amperometric hydrogen peroxide electrode and to a d5126-2 Omniscribe analog recorder from Houston Instruments (Houston, NS, USA). The test solution was contained in a thermostated cell at 25 °C under constant magnetic stirring (291/lf, Amel Instruments, Milan, Italy).

### 2.3. Flow-Immunosensor SPR

**Figure 1:**
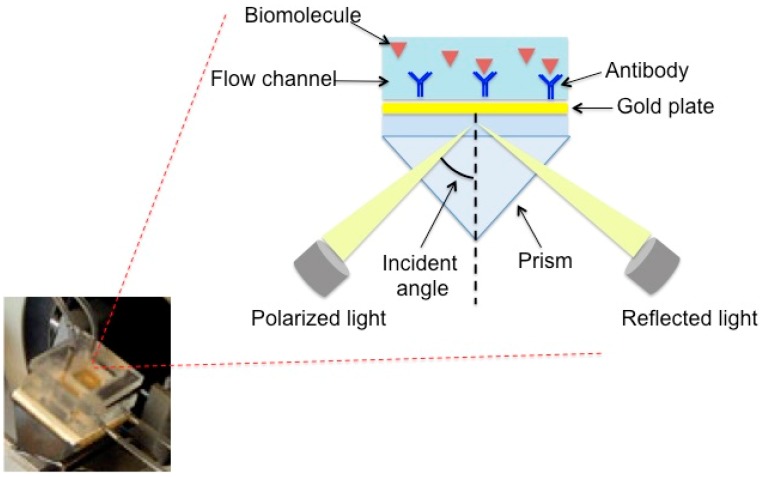
Measurement device used for surface plasmon resonance (SPR) operating in flow mode. (Of course, only the well “oriented” immobilized antibody, which can contribute to the response, has been reported).

As shown in Figure 1, the SPR flow device has a prism as the main component, installed on a rotating plate. The instrument is automatically controlled by a computer, and the samples are introduced into the cell by means of a peristaltic pump. The sensor consists of a gold sheet that is 50 nm thick and is placed on a glassy support using oil with a refractive index equal to that of the prism. To observe the SPR phenomenon, the polarized light is reflected from the wafer on a detector and monitored as a function of the resonance angle. After the formation of the complex between antibody and analyte, the SPR angle changes and this variation is proportional to the concentration of the solutions.

#### 2.3.1. Functionalization of the Gold Plate

The functionalization of the gold surface is carried out by dipping the plate in a solution containing 2 mM 1,1-mercaptoundecanoic acid (MUA) in order to form a self-assembled monolayer (SAM). A SAM structure forms spontaneously with the mercapto groups of the MUA able to form Au–S covalent bonds, while the carboxylic groups, which remain free, are available for subsequent binding with the antibody. After about 12 h, the functionalized plate is washed with ethanol and dried with nitrogen. Then, the SAM-modified gold plate is placed onto the prism surface by means of a drop oil (refractive index <2 × 10^−6^).

#### 2.3.2. Immobilization of Anti-Ampicillin

**Figure 2:**
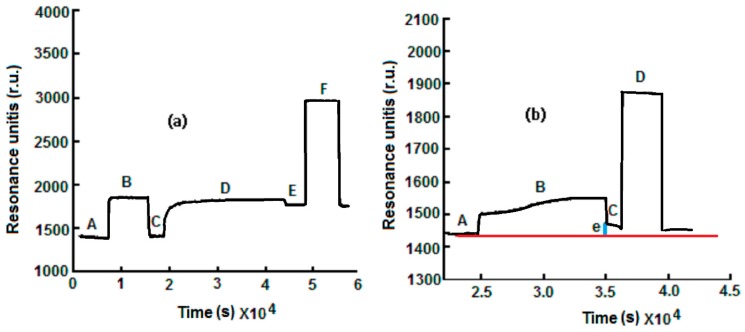
(**a**) Immobilization of anti-ampicillin: (A) baseline obtained after rehydratation of the self-assembled monolayer (SAM); (B) activation with 1-ethyl-3-(3-dimethyl-aminopropyl) carbodiimide (EDC)/N-hydroxysuccinimide (NHS) (EDC/NHS); (C) washing with buffer solution; (D) immobilization of 0.5 g·L^−1^ anti-ampicillin; (E) washing with buffer solution; (F) inactivation of non reacted –COOH groups with ethanolamine; (e = Δ in r.u.). (**b**) Example of response of anti-ampicillin SPR immunosensor towards ampicillin.

The sensorgram reported in Figure 2a is relative to the immobilization of anti-ampicillin. The first operation was the stabilization of the SPR signal with the rehydratation of the MUA by a 0.1 M flowing phosphate buffer at pH 7.4 for about 1 h. After the stabilization of the baseline (Line A, Figure 2a), a mixture of 0.5 mM ethyl (dimethylamminopropyl) carbodiimide and 0.1 mM N-hydroxysuccinimide) was allowed to flow for 15 min in order to activate the carboxylic groups of the SAM, and an increase in the signal was observed (Line B, Figure 2a).

Then, the phosphate buffer was allowed to flow to remove the excess of the EDC/NHS mixture with a lowering of the signal (Line C, Figure 2a). After the activation step, a phosphate buffer containing a concentration of 0.5 g·L^−1^ of anti-ampicillin antibodies was allowed to flow over the sensor surface for 20–30 min to achieve a covalent cross-linking of the amino reactive groups of antibodies with the aldehyde terminals (Line D, Figure 2a). Finally, after washing with the buffer solution (Line E, Figure 2a), the non-reacted, activated groups were deactivated by treatment with ethanolamine 10^−3^ M for 10 min in order to avoid non-specific adsorption (Line F, Figure 2a). As shown in Figure 2a, anti-ampicillin was effectively immobilized on the sensor surface. The baseline, after the ampicillin immobilization step, is located at an r.u. (Units of Resonance) value greater than the baseline before the antibody immobilization.

#### 2.3.3. Association of Ampicillin

For the measurement, the following sequence was used: first, the baseline (Line A, Figure 2b), in the presence of the flowing buffer (0.1 M sodium phosphate buffer, pH 7.4), was recorded, and the ampicillin solution was then allowed to flow into the cell, thus generating a signal increase (Line B, Figure 2b). When a plateau was reached, the phosphate buffer was allowed to flow to eliminate the ampicillin that was not bound to the anti-ampicillin (Line C, Figure 2b), and the Δ in r.u. values related to the ampicillin solution were determined (Dotted Line E, Figure 2b). Finally, the surface was regenerated by allowing a 0.1 mM glycine–HCl solution, pH 2.5, to flow in the SPR cell (Line D, Figure 2b), producing the dissociation of the anti-ampicillin-ampicillin complex. All the obtained Δ as units of resonance (r.u. values) were plotted as a function of the relative ampicillin concentration to obtain a calibration curve.

### 2.4. The Conventional Amperometric Immunosensor

**Figure 3:**
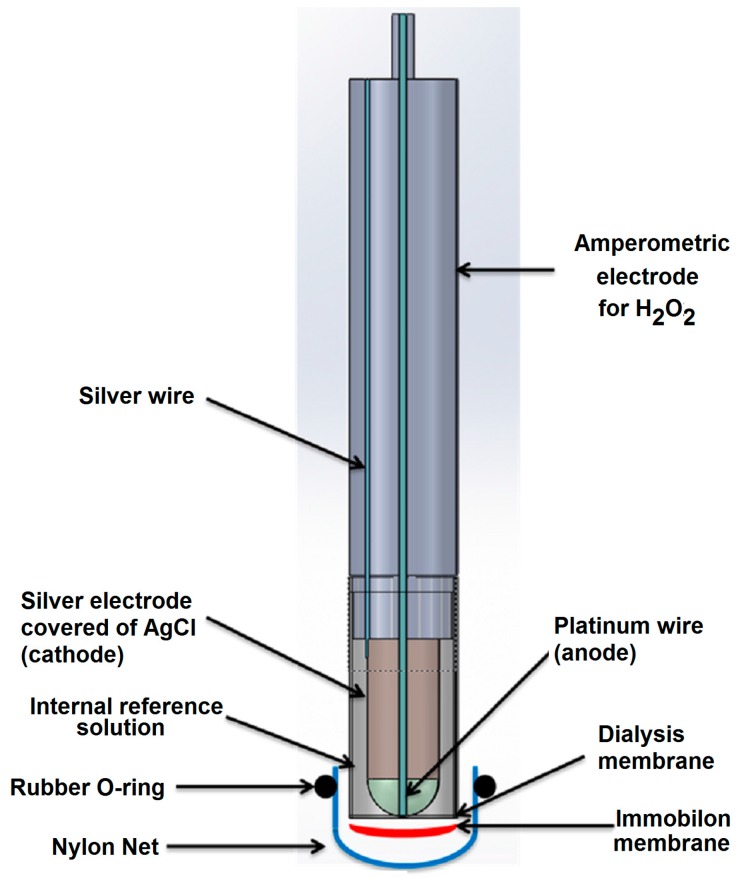
View of amperometric immunosensor for ampicillin determination using an H_2_O_2_ electrode as transducer and peroxidase as enzyme label.

The conventional amperometric immunosensor (Figure 3) was assembled using an Immobilon membrane in which the antibody or the antigen was immobilized and which overlapped a cellulose acetate membrane (0.1 mm thick) placed on the lower end of the plastic cap of an amperometric electrode for H_2_O_2_. A nylon net and a rubber O-ring were used to fix the Immobilon membrane to the head of the cap itself. The transducer used consisted of an amperometric electrode for hydrogen peroxide, with a Pt anode polarized at +0.7 V versus an Ag/AgCl/Cl^−^ cathode provided with a plastic cap filled with a 0.1 M KCl solution and screwed onto the body of the electrode. Horseradish peroxidase enzyme was used as a marker for immunocomplex detection.

#### 2.4.1. Ampicillin Biotinylation

Exactly 0.2 mL of a 1.0 mg·mL^−1^ ampicillin solution in a sodium phosphate buffer (pH 7.4; 0.1 M) was prepared. A BAC-SulfoNHS solution (5 mg·mL^−1^) was also prepared separately by dissolving 5 mg of biotinamido hexanoic acid 3-sulfo-N-hydroxysuccinimide ester in 30 µL of DMSO (dimethylsulfoxide) and adding another sodium phosphate buffer (pH 7.2; 0.1 M) for a final volume of 1 mL. Then, 10 µL of the BAC-SulfoNHS solution were immediately added to the ampicillin solution via gentle stirring, and the mixture incubated under stirring for 30 min at room temperature. The dry Sefadex G-50 resin, contained in a microspin column, was re-suspended in the column by vortexing and equilibrated with 0.2 mL of PBS (pH 7.40; 0.01 M); this buffer is needed as an equilibration buffer of the microspin G-50 column and for the elution of the labeled protein from the column. The biotinylation reaction mixture was applied to the top center of the resin, and the column was centrifuged for 5 min at 700× *g*. The purified sample was then eluted and collected at the bottom of an Eppendorf test tube. This step was repeated twice more, and a total of three fractions were collected. An ExtrAvidin peroxidase solution (20 µL, 2.0 mg·mL^−1^) was added to the collected fractions, incubated for 1 h at room temperature, and stored in a freezer at −20 °C.

#### 2.4.2. Anti-Ampicillin Immobilization on the Immobilon Membrane

The positively charged nylon Immobilon Ny^+^ membranes [22] were cut into 1 cm^2^ disks; then, 50.0 µL (3.4 × 10^−5^ M) of an anti-ampicillin solution was prepared in an Eppendorf test tube by dissolving 25 µL (10.3 mg·mL^−1^) of a standard antibody solution in 500 μL of a 0.1 M phosphate buffer, pH 7.4. This mixture was coated directly onto the surface disk membrane. The Immobilon membrane obtained was then dried at room temperature for about 24 h and stored at 4 °C.

#### 2.4.3. Ampicillin Determination with the “Competitive Format”

The Immobilon membrane, on which the anti-ampicillin was immobilized, as described in Section 2.4.2, was fixed to the head of the amperometric electrode for hydrogen peroxide. Before measurement, the immunosensor was dipped into a Tris-HCl buffer solution, 0.1 M (pH 8.0), containing 0.05% Tween-20 by weight and 2.5% BSA by weight (bovine albumin was used to minimize non-specific adsorption on the membrane).

The same competitive format for penicillin, described in detail in previous papers [22,23], was used, i.e., the ampicillin, free in solution, to be determined was allowed to compete with a fixed supply of the ampicillin labeled with biotin–avidin–peroxidase for the ampicillin antibody immobilized on the membrane (Immobilon) in order to produce the antigen-antibody immunocomplex.

**Figure 4:**
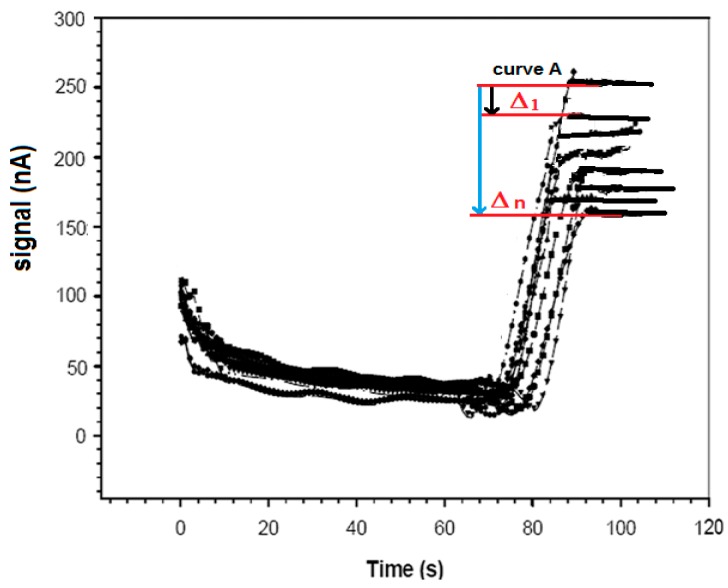
Trend in amperometric responses in the absence of peroxidase conjugated (curve A) and another curve in the presence of enzyme for H_2_O_2_, obtained by the procedure described in Section 2.4.3.

In detail, the ampicillin sample to be determined was added to 5 mL of a new phosphate buffer solution 0.1 M (pH 7.4) restored in the measurement cell, together with a fixed concentration of 50 μL (10 mg·mL^−1^) of ampicillin–biotin–avidin–peroxidase conjugated (final concentration 0.93 × 10^−6^ M). For 1 h, the enzyme-conjugated ampicillin was allowed to compete with the non-conjugated ampicillin that was free in solution that try to bind the anti-ampicillin immobilized on the Immobilon membrane. After washing with the same buffer solution to remove all unbound labeled ampicillin, the specific substrate of the enzyme, i.e., 25 μL of a 1% (v/v) H_2_O_2_ solution, was added to the renewed buffer solution in which the immunosensor was dipped, and the mixture was then stirred. The ensuing enzyme reaction was catalyzed by the enzymatic marker. The measured signal (nA) correlated directly with the ampicillin concentration to be measured. In this case, hydrogen peroxide produced a signal that increased with increasing concentrations of ampicillin free in solution. The higher the amount of ampicillin bound to the antibody immobilized on the Immobilon membrane, the lower the H_2_O_2_ consumed in the enzymatic reaction and the stronger the signal of the H_2_O_2_ amperometric electrode. The final signal was obtained by measuring the difference between this signal and that of a blank measured under the same conditions, but the latter was measured using a probe without the antibody complex immobilized on the membrane (see Figure 4).

**Figure 5:**
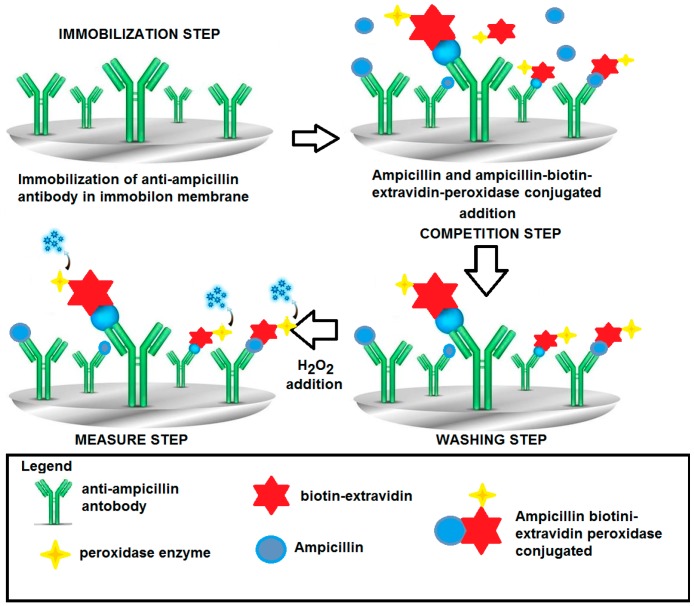
Scheme for determination of ampicillin by immunosensor using the “competitive format”; peroxidase enzyme as marker and H_2_O_2_ electrode as transducer. Test geometry: Competition between ampicillin–biotin–avidin–peroxidase conjugated and ampicillin to be measured, both free in solution, for anti-ampicillin immobilized in membrane. (Of course, only the well “oriented” immobilized antibody, which can contribute to the response, has been reported).

A calibration curve was constructed by plotting the response (in nA) on a semilogarithmic scale as a function of increasing ampicillin concentration in solution and was used to determine the unknown concentration of ampicillin contained in any sample. The schematic “competitive format” for measuring ampicillin is shown in Figure 5. The enzymatic reaction response took about 10 min. Individual measurements were performed, each time using a new membrane.

## 3. Results

**Figure 6:**
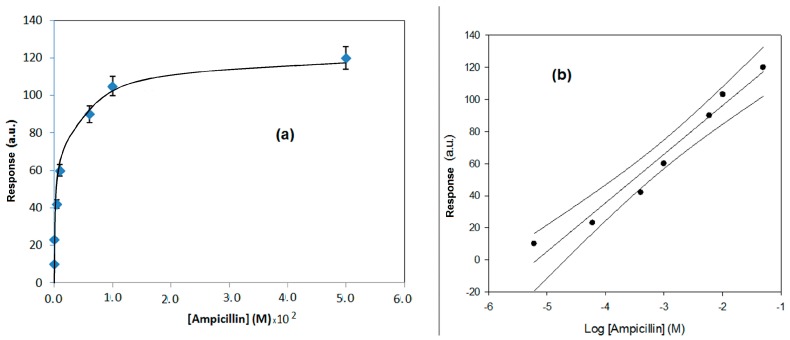
(**a**) Behavior of the response of the direct method based on flow SPR vs. concentration, for ampicillin determination. (**b**) Calibration curve using a semi-logarithmic scale and confidence interval, obtained by the direct method, based on flow SPR, for ampicillin determination.

**Figure 7:**
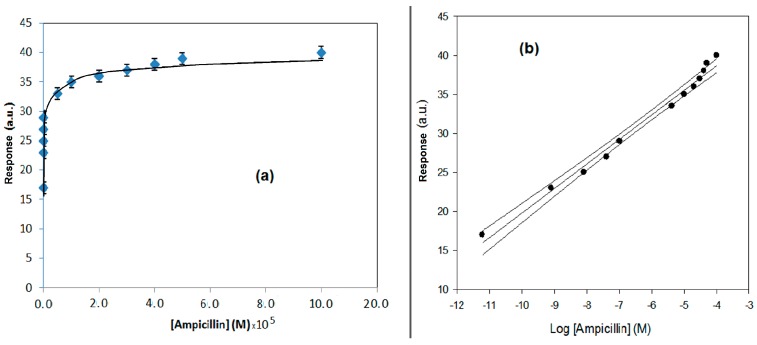
(**a**) Behavior of the conventional amperometric immunosensor response using the competitive format as a function of increasing ampicillin concentration; H_2_O_2_ electrode as transducer and peroxidase enzyme as marker. (**b**) Calibration curve for the conventional amperometric immunosensor and confidence interval using the competitive format for ampicillin determination obtained using a semilogarithmic scale.

In Figure 6a,b, the response behavior and the calibration curve, obtained using the flow SPR immunodevice, are respectively reported. In Figure 7a,b, the response behavior of the conventional amperometric immunosensor and the relative calibration curve are displayed.

In Table 1, the main analytical data found using the two immunosensors are summarized.

*K_aff_* and IC_50_ values, obtained using both the SPR device, operating in flow mode, and the conventional amperometric immunosensor, were collected and are shown in Table 2.

Selectivity data are shown in Table 3.

After both the SPR immunosensor and the ampicillin conventional amperometric immunosensor were analytically standardized, and after *K_aff_* values were evaluated and selectivity checked, both devices were used to determine the concentration of ampicillin in surface spring and river water samples and in cow milk samples (see results reported in Table 4).

The values found were also compared with similar data found in the literature [25,26,27,28], which are reported in Table 4. Furthermore, several recovery tests were carried out in the analyzed samples using both methods (see Table 5 and Table 6 respectively). All values are expressed as M of ampicillin.

We carried out a comparison between other β-lactam antibiotic sensors of every type reported in the literature in a previous work [22], wherein the amperometric competitive method was compared with 27 different methods. In that study, it was found that only two of these methods (which are more sophisticated) can reach LOD values lower than that of the conventional competitive format that we developed. Data found using other immunoassay methods are shown in Table 7, where it can be seen that our competitive format method has an LOD of about 0.087 μg·L^−1^, which is of about the same order, or better, than most immunoassay methods reported in the literature. By comparison, the direct SPR method yielded an LOD value of at least two or three decades higher than LOD values of other immunoassays. The previous developed SPR sandwich method [24] had shown an LOD five or six decades higher.

## 4. Discussion

Looking to the behavior curves of Figure 6a and Figure 7a showing the response of the immunodevices, an almost logarithmic trend can be observed in both cases. In the case of the conventional immunosensor, this trend was expected and can be easy attributed to the competitive format used, as several previous examples have tested [22,23]. On the contrary, with the SPR direct format, a linear trend was expected [24], and the found almost logarithmic trend has been surprised. We believe that the explanation must attempt to explain the antibody complex dissociation that is, as was demonstrated previously, particularly difficult in the case of ampicillin and its antibody [24]. The hydrolytic dissociation is likely also influenced from the different antigen concentration added in complex formation, such that the result of each measurement, performed at different antigen concentration in solution, depends on not only the step of formation of the antibody complex, but also the hydrolytic dissociation step of the complex, which can influence the result of the measurement, as the original baseline was probably not restored after the regeneration step.

A comparison between the main analytical data of the SPR immunosensor and those of the classical amperometric immunosensor shown in Table 1 and Table 2 indicates that better analytical results were found using the conventional immunosensor regarding sensitivity, linearity range, and LOD and *K_aff_* values, while precision is of the same order; on the contrary, the measurement time is shorter with the SPR method (Table 1) and selectivity is usually improved (see results in Table 3). On the other hand, comparing the analytical data obtained using the direct SPR method, with those that we recently obtained still employing the flow SPR method using a sandwich format [24], it is evident that the present direct method has a wider linearity range and a lower LOD value by about three decades. However, Table 1 shows that the linearity range of the present SPR method is only within about four decades and so is less wide compared with that of the conventional immune-amperometric method (which has a linearity range of about six decades). The latter, above all, is displaced towards lower concentration values. These results are in agreement with the *K_aff_* values of the two methods. In fact, the one relative to the conventional method is about four decades higher than that obtained with the SPR method; therefore, the sensitivity and LOD value of the conventional method is able to analyze all kinds of real samples, whereas the sensitivity of the SPR method is still at least useful for milk analysis, but it cannot be employed for environmental or natural water samples measurements (Table 4). Lastly, recovery data shown in Table 5 and Table 6 are not so different in the case of milk samples for both immunosensors.

Concerning selectivity, it is interesting to observe that the SPR immunosensor responds primarily to ampicillin, while the conventional one responds better to penicillin G. The use of the SPR immunosensor is therefore preferable when determining samples containing primarily ampicillin. Furthermore, Table 3 shows that the response of the conventional amperometric immunosensor to dicloxacillin, piperacillin, amoxicillin, and cefotaxime is very similar to the response to ampicillin, so the conventional device is more useful for determining the β-lactam pool of antibiotics. Additionally, in the case of the SPR immunosensor, the response to ampicillin is higher than that to the aforementioned β-lactam antibiotics.

## 5. Conclusions

The purpose of this research was to compare the direct determination of ampicillin, using the SPR technique operating in flow conditions, with results found with a conventional competitive immunosensor. The results were perhaps predictable, but we clearly establish here that, in an experimental way, at least for the determination of comparatively low molecular weight compounds (as in the case of β-lactam antibiotics), the amperometric conventional method, compared to direct methods that easily attain data with the SPR transducer, provides the best results from an analytical point of view. Nevertheless, experimental results highlight the great selectivity of the SPR immunosensor in a concentration range from 10^−6^ M to 10^−2^ M. Another important positive aspect of the SPR method is the analysis time. In fact, a direct determination of ampicillin was performed in a shorter time. On the other hand, the conventional immunosensor method has a lower LOD and a wide linear range, but its analysis time is very long (about 1 h) due to the competitive format. Further, this competitive immunosensor exhibits low selectivity towards other β-lactam antibiotics, but good selectivity towards antibiotics that do not belong to the same class. This sensor therefore can be useful for the determination of the “pool” of antibiotics of the β-lactam class in common real matrices, such as river or surface natural water, which can be more or less polluted by these types of antibiotics. It is clear that, on the basis of these results, the conventional competitive device, compared to the SPR device that has a very high LOD for this kind of application, is preferred for its much lower LOD values when perform β-lactam antibiotics analysis on real natural water samples, even though recovery data on milk samples obtained with the standard addition method yielded reasonable results with the SPR immunosensor. The real advantages of the SPR method (which is a remarkable advantage) consists in a drastic reduction in analysis time, as well as in the exemplification of the measurement format. In addition, the SPR immunosensor, compared with the conventional immunosensor (that is, for instance, more selective towards penicillin than to ampicillin), is more selective towards ampicillin compared to all other beta-lactam antibiotics.

## Figures and Tables

**Table 1:** The main analytical data found using two immunosensors for the determination of ampicillin.

Method	Determination of Ampicillin with SPR Immunosensor	Determination of Ampicillin with Conventional Amperometric Immunosensor
Geometry of the test	Direct measurement between ampicillin free in solution and anti-ampicillin immobilized	Competition between ampicillin–biotin–avidin–peroxidase conjugated and ampicillin, both free in solution, for anti-ampicillin immobilized in membrane.
Regression equation (Y = a.u., X = M) Confidence level (1 − α) = 0.95	Y = 13.03 (±0.78) log X + 162.8 (±6.2) (*n* − ν) = 6; (*t* = 2.23)	Y = 50.1 (±2.3) log X + 1.3 (±0.1) (*n* − ν) = 16; (*t* = 2.23)
Linear range (M)	2.5 × 10^−6^–3.0 × 10^−2^	5.0 × 10^−1^–1 × 10^−4^
Correlation coefficient	0.9830	0.9806
Repeatability of each measurement as RSD%	4.8 (*n* = 3)	5.0 (*n* = 5)
Repeatability of the measurement (as pooled SD%)	6.9	7.5
Low detection limit (LOD) (M)	1.0 × 10^−6^	2.5 × 10^−1^
Instrumental response time (min)	≅15	≅10
Measurement time (min)	≤20	≅75

**Table 2:** IC_50_ and *k_aff_* values for the ampicillin obtained by the SPR immunosensor, and the values found using the conventional amperometric immunosensor.

Method	IC_50_ *n* = 5; RSD% ≤ 5 (M)	*K_aff_* *n* = 5; RSD% ≤ 5 (M^−1^)
Amperometric ampicillin competitive format device	2.7 × 10^−8^	3.7 × 10^7^
Direct SPR immunosensor for ampicillin	5.0 × 10^−4^	2.0 × 10^3^

**Table 3:** Percent cross selectivity values for several common antibiotics.

Antibiotics	%Response of Several Antibiotics RSD% *≤ 5*.0	
	SPR Flow-Immunosensor ^1^	Conventional Amperometric Immunosensor ^2^
Ampicillin	100.0	68.9
Potassium penicillin G	41.0	100
Dicloxacillin	35.5	70.7
Piperacillin	29.0	61.5
Amoxicillin	23.5	52.0
Ceftriaxone	18.0	-
Cefalexin	17.6	38.7
Cefotaxime	-	48.5
Fosfomycin	11.7	39.0
Neomycin	11.7	24.2
Erythromycin	-	30.5
Bacitracin	-	2.3
Ac. amino 6-penicillamic	-	18.0

^1^ Ampicillin checked as 100%. ^2^ Penicillin G checked as 100%.

**Table 4:** “Pool” of β-lactam antibiotic concentration, expressed as M (of ampicillin), found in bovine milk and river and spring surface water samples using the direct SPR immunosensor and the amperometric immunosensor using a competitive format.

Matrix	Concentration of β-lactam Antibiotic “Pool” Found in Samples Using SPR Immunosensor *n* = 3; RSD% ≤ 5.5 (M)	Concentration of β-lactam Antibiotic “Pool” Found in Samples Using Conventional Immunosensor *n* = 5; RSD% ≤ 5.5 (M)	Values of β-Lactam Antibiotics Reported in Literature	References
Bovine milk (first sample)	≈1 × 10^−6^	1.30 × 10^−6^	(0.4*–*17.0) × 10^−6^ (0.6*–*17.5) × 10^−6^	[25] [11]
Bovine milk (second sample)	≈1 × 10^−6^	2.40 × 10^−6^	(0.4*–*17.0) × 10^−6^ (0.6*–*17.5) × 10^−6^	[25] [11]
“SACCO” river water sample	≤1 × 10^−6^	1.45 × 10^−7^	(0.9*–*60.0) × 10^−8^ (0.1*–*3.0) × 10^−8^	[26] [27]
Spring surface water sample	≤1 × 10^−6^	8.30 × 10^−8^	(0.9*–*60.0) × 10^−8^ (0.1*–*3.0) × 10^−8^ 1.0 × 10^−9^	[26] [27] [28]

**Table 5:** Recovery tests for ampicillin using the SPR immunosensor in real samples of bovine milk and of river and spring water samples; values expressed as M (of ampicillin).

Matrix	Concentration of β-Lactam Antibiotic “Pool” Found in Samples Using SPR Immunosensor *n* = 3; RSD% ≤ 5.5 (M)	Concentration of Ampicillin Added in the Samples (M)	Nominal Concentration of β-Lactam Antibiotic *n* = 3; RSD% ≤ 5.5 (M)	Experimental Concentration Found in Spiked Samples Using SPR Immunosensor *n* = 3; RSD% ≤ 5.5 (M)	%Recovery Using SPR Immunosensor RSD% ≤ 5.5
Bovine milk (first sample)	≈1 × 10^−6^	4.00 × 10^−6^	5.00 × 10^−6^	4.81 × 10^−6^	96.2%
Bovine milk (second sample)	≈1 × 10^−6^	4.00 × 10^−6^	5.00 × 10^−6^	4.88 × 10^−6^	97.6%
“SACCO” River water	≈1 × 10^−6^	4.00 × 10^−6^	5.00 × 10^−6^	4.75 × 10^−6^	95.0%
Standard Spring water	≈1 × 10^−6^	4.00 × 10^−6^	5.00 × 10^−6^	4.83 × 10^−6^	96.6%

**Table 6:** Recovery tests for ampicillin using the conventional amperometric immunosensor in real samples of bovine milk and river and spring surface water samples; values expressed as M (of ampicillin).

Real Matrix	Concentration of β-Lactam Antibiotic “Pool” Found in Samples Using Conventional Amperometric Immunosensor. *n* = 5; RSD% ≤ 5.5 (M)	Concentration of Ampicillin Added in the Samples (M)	Nominal Concentration of β-Lactam Antibiotic *n* = 5; RSD% ≤ 5.5 (M)	Experimental Concentration Found in Spiked Samples Using Amperometric Immunosensor *n* = 5; RSD% ≤ 5.5 (M)	%Recovery Using Amperometric Immunosensor RSD% ≤ 5.5
Bovine milk (first sample)	1.30 × 10^−6^	1.00 × 10^−6^	2.30 × 10^−6^	2.28 × 10^−6^	99.1%
Bovine milk (second sample)	2.40 × 10^−6^	1.50 × 10^−6^	3.90 × 10^−6^	3.75 × 10^−6^	96.2%
“SACCO” river water sample	1.45 × 10^−7^	1.05 × 10^−7^	2.50 × 10^−7^	2.48 × 10^−7^	99.2%
Spring surface water sample	8.30 × 10^−8^	0.7 × 10^−8^	9.00 × 10^−8^	8.70 × 10^−8^	96.6%

**Table 7:** Data found using other immunoassay methods and immunosensor methods that we have developed.

Method Assay	Range (μg·L^−1^)	LOD (μg·L^−1^)	Reference
EIA	/	4.6	[29]
ELISA (enzyme-linked immunosorbent assay)	10–50	/	[30]
ELISA (enzyme-linked immunosorbent assay)	/	2.97	[31]
Fluorescent immunoassay	6.0–191	2.4	[17]
Solid-Phase Fluorescence Immunoassay	/	50	[32]
Fluorescent Immunoassay	2.0–10	2.9	[20]
Solid-Phase Fluorescence Immunoassay	/	1.0	[33]
Our competitive format amperometric method	0.17–34.9 × 10^3^	0.087	This paper
Our direct SPR method	875–10.5 × 10^6^	350	This paper
Our sandwich Flow SPR method	350 × 10^3^–34.9 × 10^6^	350 × 10^3^	[24]
/	/	4.0 (in milk)	[34]
